# Antibacterial and cytotoxic activities of naphthoquinone pigments from Onosma visianii Clem

**DOI:** 10.17179/excli2016-762

**Published:** 2017-02-16

**Authors:** Milena D. Vukic, Nenad L. Vukovic, Gorica T. Djelic, Suzana Lj. Popovic, Milan M. Zaric, Dejan D. Baskic, Gordana B. Krstic, Vele V. Tesevic, Miroslava M. Kacaniova

**Affiliations:** 1 University of Kragujevac, Faculty of Science, Department of Chemistry, R. Domanovica 12, 34000 Kragujevac, Serbia; 2 University of Kragujevac, Faculty of Science, Department of Biology and Ecology, R. Domanovica 12, 34000 Kragujevac, Serbia; 3 University of Kragujevac, Faculty of Medical Sciences, Centre for Molecular Medicine and Stem Cell Research, 34000 Kragujevac, Serbia; 4 University of Kragujevac, Faculty of Medical Sciences, Department of Biochemistry, 34000 Kragujevac, Serbia; 5 University of Belgrade, Faculty of Chemistry, Belgrade, Serbia; 6 University of Agriculture in Nitra, Department of Microbiology, Faculty of Biotechnology and Food Science, Slovak Nitra, Slovakia

**Keywords:** Onosma visianii, naphthoquinones, antibacterial activity, cytotoxic activity

## Abstract

In this study, the antibacterial and cytotoxic activities of isolated compounds from the roots of *Onosma visianii* were investigated. By using different chromatographic techniques and appropriate spectroscopic methods, the seven naphthoquinones were described: deoxyshikonin (***1***), isobutyrylshikonin (***2***), α-methylbutyrylshikonin (***3***), acetylshikonin (***4***), β-hydroxyisovalerylshikonin (***5***), 5,8-*O*-dimethyl isobutyrylshikonin (***6***) and 5,8-*O*-dimethyl deoxyshikonin (***7***). Among the tested compounds, ***3*** and ***4*** exhibited the highest antibacterial activities toward all tested bacterial species (MIC_50_ and MIC_90 _for gram positive bacteria: 6.40 μg/mL-12.79 μg/mL and 6.82 μg/mL-13.60 μg/mL, respectively; for gram negative bacteria: 4.27 μg/mL-8.53 μg/mL and 4.77 μg/mL-9.54 μg/mL, respectively). Also, naphthoquinones ***3*** and ***4*** exhibited strong cytotoxic activity against MDA-MB-231 cells (IC_50_ values 86.0 μg/mL and 80.2 μg/mL, respectively), while compounds ***1***,*** 3***, ***4*** and ***5*** significantly decreased viability of HCT116 cells (IC_50_ values of 97.8 μg/mL, 15.2 μg/mL, 24.6 μg/mL and 30.9 μg/mL, respectively). Our results indicated that all tested naphthoquinone pigments are potential candidates for clinical uses as antibacterial and cytotoxic agents.

## Introduction

The emergence of antibiotic resistance presents one of the most important challenges for the pharmaceutical industry and the healthcare sector both in the developing and developed countries. The inadequate and excessive use of antibiotics, significantly diminishes the efficacy of current drugs, at the same time demanding new drugs against multi-drug resistant bacteria (Gyles, 2011[11]). Another major health problem is cancer. It affects about two hundred types of cell lines, causing lack of control of the cell proliferation and differentiation while invading various tissues and organs. There are many difficulties in the treatment of these diseases, especially in terms of drug resistance, toxicity, and low specificity. Although the official medicine has shown effective results, herbal medicinal products successfully act on some kinds of these ailments.

The genus *Onosma* L. (*Boraginaceae*) contains about 150 species, distributed mainly in the Mediterranean region and Central Asia (El-Shazly et al., 2003[10]).

The roots of *Onosma sericeum *Willd*.*, *Onosma microcarpum *DC., *Onosma argentata *Hub.-Mor*. *and *Onosma armeniaca *Klokov have been widely used in traditional medicine for the treatment of wounds, burns, dyspnea, hoarseness, hemorrhoids, abdominal aches and stomach ulcers (Sezik et al., 1997[24]; Davis, 1988[8]; Cadirci et al., 2007[5]).

Phytochemical investigation of the roots of genus* Onosma* has led to the discovery of a series of shikonin and alkannin derivatives, as compounds which exhibit a wide spectrum of biological properties, like wound healing, antiinflammatory, antitumor, antimicrobial and antithrombotic properties (Papageorgiou et al., 1999[23], 2008[22]; Wang et al., 2015[26]; Naz et al., 2006[18]). On the other hand, literature reported that aerial parts contain pyrrolizidine alkaloids (El-Shazly et al., 2003[10]; Damianakos et al., 2013[7]) and phenolic compounds (Mellidis et al., 1993[15]).

*Onosma visianii* Clem (*Boraginaceae*) is a biennial to perennial plant which inhabits areas of Balkan Peninsula and Southeast Europe. The point of view of the chemical composition, *O. visianii* is little investigated. Except that it contains shikonin (Shcherbanovskii, 1971[25]), other literature data relating to chemical composition and pharmacological activity do not exist.

We herein report for the first time isolation and structural characterization of these naphthoquinones from the roots of *O. visianii*. Also, antibacterial activity against ten bacterial species, as well as cytotoxic activity against MDA-MB-231 and HCT116 tumor cell lines was described.

## Materials and Methods

### Chemicals

Petroleum ether (boiling point ranges: 40 °C-60 °C), methylene chloride, ethyl acetate, deuterochloroform (CDCl_3_), dimethyl sulfoxide (DMSO) and HPLC grade methanol were obtained from Sigma-Aldrich (Steinheim, Germany). Water was treated in a Milli-Q water purification system (TGI Pure Water Systems, Brea, CA, USA). Mueller Hinton agar was obtained from Merck (Germany). Nutrient liquid medium, Mueller Hinton broth was purchased from Biolife (Italy). 3-(4,5-Dimethylthiazol-2-yl)-2,5-diphenyltetrazolium bromide (MTT) was obtained from SERVA (Heidelberg, Germany). Annexin V-FITC/7-AAD Kit was purchased from Beckman Coulter (USA). Standard antibiotics, Cisplatin, Phosphate-buffered saline (PBS), Ribonuclease A (RNAse A), fetal bovine serum (FBS) and 7-Aminoactinomycin D (7-AAD) were obtained from Sigma-Aldrich (Steinheim, Germany). 

### Instrumentation

Microanalysis of carbon and hydrogen was carried out with a Carlo Erba 1106 microanalyser. Infrared spectra were run on Thermo Scientific Nicolet 6700 FT-IR spectrometer (4000-400 cm^-1^) and Perkin-Elmer FT-IR Spectrometer Spectrum One (KBr disc, *ν* in cm^-1^). The NMR spectra were recorded on a Varian Gemini 200 spectrometer (^1^H at 200 MHz and ^13^C at 50 MHz) and a BrukerAvance III 500 spectrometer (^1^H at 500.26 MHz and ^13^C at 125.80 MHz), solvent CDCl_3_, TMS internal standard. Chemical shifts were given in δ (ppm), *J*-coupling constants in Hertz (Hz), abbreviations: s-singlet, d-doublet, dd-doublet of doublet, t-triplet, m-multiplet, br s-broadend singlet. Low resolution mass spectra were recorded on an Agilent 5973 mass spectrometer (Santa Clara, CA, USA). High resolution mass spectra were obtained on a Agilent 6210 Time of Flight (TOF) mass spectrometer (Agilent Technologies, Santa Clara, CA, USA) and linear-trap quadrupole (LTQ) orbitrap hybrid mass spectrometer equipped with a heated-electrospray ionization probe (HESI-II, ThermoFisher Scientific, Bremen, Germany). [α]_D_ values were measured in dichloromethane using a Perkin Elmer 341 polarimeter. UV/VIS spectra were recorded on an Agilent Cary 300 UV-VIS spectrophotometer (Agilent Technologies, Palo Alto, USA). MALDI TOF MS (Matrix Assisted Laser Desorption Ionization-Time of Flight Mass Spectrometry) Biotyper (Bruker Daltonics, Billerica, USA) was used for confirmation of test bacteria. Semi preparative high performance liquid chromatography was performed on Agilent 1100 Series liquid chromatograph (Agilent Technologies, Santa Clara, CA, USA) equipped with diode array detector (DAD; λ=520 nm, λ=450 nm), autosampler, and fraction collector; conditions: injection volume, 600 μL (2 mg/mL, methanol); column Zorbax Eclipse XDB C18 (250mm x 9.4 mm; 5 μm); mobile phase (6 mL/min), water (40 %) and methanol (60 %). Silica gel 60 (Merck, 70-230 mesh) was used for column chromatography. Preparative TLC was performed by using silica gel P/UV254 with CaSO_4_ (Machery-Nagel, Germany, 2 mm layer of adsorbent). Analytical TLC was performed on silica gel (Silica gel 60, layer 0.20 mm, Alugram Sil G, Mashery-Nagel, Germany). 

### Plant material

*O. visianii* Clem roots were collected (June 2015) in the region of mountain Rumija (southern Montenegro, altitude 650 m, 42º 06' 10'' N, 19º 11' 37'' E). A voucher herbarium specimen was deposited at the Department of Botany, Faculty of Biology, University of Belgrade, Serbia (17130, BEOU).

### Extraction method

Dried roots (135 g) of *O. visianii* were powdered and extracted at room temperature three times with a (1500 mL, 24 h) petroleum ether-methylene chloride mixture (1:1). The combined extracts were evaporated under reduced pressure to give concentrated extract as red-brown semi-viscous residue (6.5 g). 

### General procedure for isolation of compounds 

The petroleum ether-methylene chloride extract (6 g) was subjected to silica gel column chromatography. The column was eluted with petroleum ether (100 %), followed by an increasing gradient of ethyl acetate starting from 1 % up to 30 %. Twenty-nine collected fractions were analyzed on TLC plates using petroleum ether:methylene chloride (97:3 and 95:5) and petroleum ether:ethyl acetate (95:5) solvent systems. Similar fractions were combined (***F1***-***11***, ***F12***-***15***, ***F16***-***20***, ***F21***-***23*** and ***F24***-***29***) and further subjected to preparative TLC. Used mobile phases: for ***F1***-***11*** and ***F12***-***15*** petroleum ether:ethyl acetate (8:2), for ***F16***-***20*** and ***F21***-***23*** petroleum ether:ethyl acetate (7:3), for ***F24***-***29*** petroleum ether:ethyl acetate (6:4). After TLC examinations, compounds ***1*** and ***2*** were obtained from ***F1***-***11***, ***2*** and ***3*** were obtained from ***F12***-***15***, from ***F16***-***20*** ***3***, ***4*** and ***5***, from ***F21***-***23*** was isolated compound ***5***, while ***F24***-***29*** provided compounds ***6*** and ***7***. In order to obtain high purity, all isolated compounds were subjected to semi preparative HPLC on Zorbax Eclipse XDB C18 reversed phase column with isocratic elution of mixture water and methanol (40:60). 

### Spectroscopic data of isolated compounds 1-7

#### Deoxyshikonin 1 

(5,8-dihydroxy-2-(4-methylpent-3-enyl)naphthalene-1,4-dione). Red purple solid. UV/VIS (MeOH, nm) λ_max_ (ε) 552 (3.33), 515 (3.41), 486 (3.34), 277 (3.80), 216 (4.47); IR (KBr, cm^-1^) 3650-3160 (OH), 3055 (=CH), 2923 (CH), 1621, 1573 and 1457 (C=C), 1222 and 1102 (C-O); ^1^H NMR (500 MHz, CDCl_3_, TMS, ppm) δ 1.60 (3H, s, H-16), 1.70 (3H, s, H-15), 2.28 (2H, m, H-11), 2.60 (2H, m, H-12a, H-12b), 5.13 (1H, t, *J*=7.1 Hz, H-13), 6.84 (1H, s, H-3), 7.21 (2H, s, H-6 and H-7), 12.48 (1H, s, OH-5) and 12.64 (1H, s, OH-8); ^13^C NMR (125 MHz, CDCl_3_, TMS, ppm) δ 17.9 (C-16), 25.7 (C-15), 26.4 (C-12), 29.7 (C-11), 111.6 (C-9), 111.8 (C-10), 122.3 (C-13), 130.7 (C-6), 131.0 (C-7), 133.5 (C-14), 134.5 (C-3), 151.5 (C-2), 161.9 (C-5 or C-8), 162.6 (C-8 or C-5), 183.2 (C-1 or C-4) and 183.3 (C-4 or C-1); HRESIMS *m/z *[M-H]^-^=271.0956 (error -3.340 ppm; calc to C_16_H_15_O_4 _*m/z *271.0970).

#### Isobutyrylshikonin 2 

[(+)-1-(5,8-dihydroxy-1,4-dioxo-1,4-dihydronaphthalen-2-yl)-4-methylpent-3-enyl isobutyrate]. Red purple solid. [α]_D_^25^ (dichloromethane, C=0.00121) +321. UV/VIS (MeOH, nm) λ_max_ (ε) 556 (3.21), 518 (3.48), 489 (3.49), 275 (3.81), 217 (4.73); IR (KBr, cm^-1^) 3680-3630 (OH), 3074 and 3048 (=CH), 2965, 2925 and 2853 (CH), 1735 (C=O from COOCH(CH_3_)_2_), 1607, 1569 and 1457 (C=C), 1238, 1213, 1146 and 1116 (C-O); ^1^H NMR (500 MHz, CDCl_3_, TMS, ppm) δ 1.20 (3H, d, *J*=7.0 Hz, H-3'), 1.22 (3H, d, *J*=7.0 Hz, H-4'), 1.58 (3H, s, H-16), 1.69 (3H, s, H-15), 2.47 (1H, m, H-12a), 2.62 (1H, m, H-12b), 2.64 (1H, m, H-2'), 5.12 (1H, t, *J*=7.5 Hz, H-13), 6.02 (1H, dd, *J*=7.2 Hz, *J*=4.5 Hz, H-11), 6.97 (1H, s, H-3), 7.18 (2H, s, H-6 and H-7), 12.42 (1H, s, OH-5) and 12.58 (1H, s, OH-8); ^13^C NMR (125 MHz, CDCl_3_, TMS, ppm) 17.9 (C-16), 18.9 (C-3' and C-4'), 25.7 (C-15), 32.8 (C-12), 33.9 (C-2'), 68.8 (C-11), 111.4 (C-9), 111.8 (C-10), 117.7 (C-13), 131.3 (C-3), 132.5 (C-6), 132.8 (C-7), 135.9 (C-14), 148.5 (C-2), 166.6 (C-5 or C-8), 167.3 (C-8 or C-5), 175.7 (C-1'), 176.7 (C-1 or C-4) and 178.3 (C-4 or C-1); HRESIMS *m/z *[M-H]^-^=357.1320 (error -3.654 ppm; calc to C_20_H_21_O_6 _*m/z *357.1338).

#### α-Methylbutyrylshikonin 3 

[(+)-1-(5,8-dihydroxy-1,4-dioxo-1,4-dihydronaphthalen-2-yl)-4-methylpent-3-enyl 2-methylbutanoate]. Red purple solid. [α]_D_^25^ (dichloromethane, C=0.00121) +221. UV/VIS (MeOH, nm) λ_max_ (ε) 557 (3.20), 518 (3.47), 489 (3.38), 275 (3.87), 216 (4.53); IR (KBr, cm^-1^) 3660-3622 (OH), 3041 (=CH), 2970, 2932 and 2878 (CH), 1742 (C=O from COOCH(CH_3_)CH_2_CH_3_), 1610, 1571 and 1455 (C=C), 1232, 1204, 1148 and 1113 (C-O); ^1^H NMR (500 MHz, CDCl_3_, TMS, ppm) δ 0.93 (3H, t, *J*=7.3 Hz, H-4'), 1.17 (3H, d, *J*=7.0 Hz, H-5'), 1.53 (2H, m, H-3'), 1.59 (3H, s, H-16), 1.69 (3H, s, H-15), 2.27 (1H, m, H-2'), 2.47 (1H, m, H-12a), 2.61 (1H, m, H-12b), 5.13 (1H, t, *J*=7.0 Hz, H-13), 6.04 (1H, dd, *J*=7.4 Hz, *J*=4.3 Hz, H-11), 6.98 (1H, s, H-3), 7.18 (2H, s, H-6 and H-7), 12.42 (1H, s, OH-5) and 12.58 (1H, s, OH-8); ^13^C NMR (125 MHz, CDCl_3_, TMS, ppm) δ 11.4 (C-4'), 17.9 (C-16), 18.8 (C-5'), 25.7 (C-15), 26.7 (C-3'), 33.0 (C-12), 41.0 (C-2'), 69.0 (C-11), 111.6 (C-9), 111.8 (C-10), 117.8 (C-13), 131.3 (C-2), 132.6 (C-6), 132. 8 (C-7), 135.9 (C-14), 148.6 (C-3), 166.9 (C-5 or C-8), 167.4 (C-8 or C-5), 175.3 (C-1'), 176.7 (C-1 or C-4), and 178.3 (C-4 or C-1); HRESIMS *m/z *[M-H]^-^=371.1473 (error -4.405 ppm; calc to C_21_H_23_O_6 _*m/z *371.1495).

#### Acetylshikonin 4 

[(+)-1-(5,8-dihydroxy-1,4-dioxo-1,4-dihydronaphthalen-2-yl)-4-methylpent-3-enyl acetate]. Red purple solid. [α]_D_^25^ (dichloromethane, C=0.00135) +692. UV/VIS (MeOH, nm) λ_max_ (ε) 556 (3.21), 518 (3.48), 490 (3.41), 273 (3.89), 215 (4.53); IR (KBr, cm^-1^) 3650-3620 (OH), 3065 (=CH), 2921 and 2859 (CH), 1732 (C=O from COOCH_3_), 1604, 1575 and 1454 (C=C), 1236, 1209, 1114 and 1049 (C-O); ^1^H NMR (500 MHz, CDCl_3_, TMS, ppm) δ 1.58 (3H, s, H-16), 1.69 (3H, s, H-15), 2.14 (3H, s, H-2'), 2.46 (1H, m, H-12a), 2.61 (1H, m, H-12b), 5.12 (1H, t, *J*=6.1 Hz, H-13), 6.02 (1H, dd, *J*=7.4 Hz, *J*=4.2 Hz, H-11), 7.00 (1H, s, H-3), 7.18 (2H, s, H-6 and H-7), 12.43 (1H, s, OH-5) and 12.58 (1H, s, OH-8); ^13^C NMR (125 MHz, CDCl_3_, TMS, ppm) δ 17.9 (C-16), 20.9 (C-2'), 25.7 (C-15), 32.8 (C-12), 69.5 (C-11), 111.5 (C-9), 111.8 (C-10), 117.7 (C-13), 131.4 (C-2), 132.7 (C-6), 132.9 (C-7), 136.1 (C-14), 148.1 (C-3), 166.9 (C-5 or C-8), 167.4 (C-8 or C-5), 169.6 (C-1'), 176.8 (C-1 or C-4) and 178.2 (C-4 or C-1); HRESIMS *m/z *[M-H]^-^=329.1004 (error -4.663 ppm; calc to C_18_H_17_O_6 _*m/z *329.1025).

#### β-Hydroxyisovalerylshikonin 5 

[(+)-1-(5,8-dihydroxy-1,4-dioxo-1,4-dihydronaphthalen-2-yl)-4-methylpent-3-enyl 3-hydroxy-3-methylbutanoate]. Red purple solid. [α]_D_^25^ (dichloromethane, C=0.00111) +91. UV/VIS (MeOH, nm) λ_max_ (ε) 556 (3.19), 518 (3.48), 489 (3.40), 276 (3.97), 217 (4.54). IR (KBr, cm^-1^) 3680-3620 (OH), 3043 (=CH), 2974, 2927 and 2853 (CH), 1737 (C=O from COOCH_2_C(CH_3_)_2_OH), 1612, 1571 and 1455 (C=C), 1264, 1204, 1111 and 1154 (C-O); ^1^H NMR (500 MHz, CDCl_3_, TMS, ppm) δ 1.31 (6H, s, H-4', H-5'), 1.59 (3H, s, H-16), 1.69 (3H, s, H-15), 2.50 (1H, m, H-12a), 2.59 (3H, s, H-2'), 2.63 (1H, m, H-12b), 3.26 (1H, br s, C-3'-OH), 5.12 (1H, t, *J*=6.7 Hz, H-13), 6.10 (1H, dd, *J*=7.8 Hz, *J*=4.2 Hz, H-11), 7.03 (1H, s, H-3), 7.18 (2H, s, H-6 and H-7), 12.41 (1H, s, OH-5) and 12.60 (1H, s, OH-8); ^13^C NMR (125 MHz, CDCl_3_, TMS, ppm) δ 18.3 (C-16), 25.7 (C-15), 29.2 (C-4'), 29.5 (C-5'), 33.1 (C-12), 46.5 (C-2'), 69.1 (C-3'), 69.8 (C-11), 111.5 (C-9), 111.8 (C-10), 117.7 (C-13), 131.3 (C-2), 133.1 (C-6), 133.3 (C-7), 136.4 (C-14), 147.5 (C-3), 168.2 (C-5 or C-8), 168.7 (C-8 or C-5), 171.6 (C-1'), 175.3 (C-1 or C-4) and 176.9 (C-4 or C-1); HRESIMS *m/z *[M-H]^-^=387.1419 (error -4.958 ppm; calc to C_21_H_23_O_7 _*m/z *387.1444).

#### 5,8-O-Dimethyl isobutyrylshikonin 6 

[(+)-1-(5,8-dimethoxy-1,4-dioxo-1,4-dihydronaphthalen-2-yl)-4-methylpent-3-enyl isobutyrate]. Orange oil**. **[α]_D_^25^ (dichloromethane, C=0.00111) +289. UV/VIS (MeOH, nm) λ_max_ (ε) 452 (3.45), 259 (4.11), 215 (4.79); IR (KBr, cm^-1^) 3044 (=CH), 2919 and 2847 (CH), 1732 (C=O from COOCH(CH_3_)_2_), 1650 and 1457 (C=C), 1278, 1155 and 1058 (C-O); ^1^H NMR (500 MHz, CDCl_3_, TMS, ppm) δ 1.18 (3H, d, *J*=4.0 Hz, H-3'), 1.19 (3H, d, *J*=4.0 Hz, H-4'), 1.57 (3H, s, H-16), 1.66 (3H, s, H-15), 2.44 (1H, m, H-12a), 2.60 (1H, m, H-12b), 3.96 (6H, s, C-5-OCH_3_, C-8-OCH_3_), 5.12 (1H, t, *J*=7.5 Hz, H-13), 5.90 (1H, dd, *J*=7.5 Hz, *J*=4.5 Hz, H-11), 6.65 (1H, s, H-3), 7.31 (2H, s, H-6 and H-7); ^13^C NMR (125 MHz, CDCl_3_, TMS, ppm) δ 17.9 (C-16), 18.9 (C-3' and C-4'), 20.9 (C-2'), 25.7, (C-15), 32.8 (C-12), 56.8 (C-5-OCH_3_ or C-8-OCH_3_), 56.9 (C-8-OCH_3_ or C-5-OCH_3_), 69.7 (C-11), 118.1 (C-13), 120.1 (C-7), 120.6 (C-9, C-10), 131.3 (C-3), 133.3 (C-6), 135.6 (C-14), 148.3 (C-2), 153.0 (C-5 or C-8), 154.0 (C-8 or C-5), 169.5 (C-1'), 183.3 (C-1 or C-4), 184.5 (C-4 or C-1); HRESIMS *m/z *[M+H]^+^=387.1824 (error 4.226 ppm; calc to C_22_H_27_O_6 _*m/z *387.1808).

#### 5,8-O-Dimethyl deoxyshikonin 7 

(5,8-dimethoxy-2-(4-methylpent-3-enyl)naphthalene-1,4-dione). Dark orange oil**. **UV/VIS (MeOH, nm) λ_max_ (ε) 443 (3.33), 259 (4.21), 216 (4.75); IR (KBr, cm^-1^) 3054 (=CH), 2923 and 2823 (CH), 1644 and 1448 (C=C), 1278, 1207, 1046 and 1018 (C-O); ^1^H NMR (500 MHz, CDCl_3_, TMS, ppm) δ 1.58 (3H, s, H-16), 1.67 (3H, s, H-15), 2.24 (1H, m, H-12a), 2.52 (3H, m, H-11, H-12b), 3.95 (6H, s, C-5-OCH_3_, C-8-OCH_3_), 5.12 (1H, t, *J*=6.4, Hz, H-13), 6.60 (1H, s, H-3), 7.30 (2H, s, H-6 and H-7); ^13^C NMR (125 MHz, CDCl_3_, TMS, ppm) δ 17.9 (C-16), 25.8, (C-15), 32.8 (C-12), 56.8 (C-5-OCH_3_ or C-8-OCH_3_), 56.9 (C-8-OCH_3_ or C-5-OCH_3_), 69.7 (C-11), 118.1 (C-13), 120.1 (C-7), 120.6 (C-9, C-10), 133.3 (C-6), 134.5 (C-3), 135.6 (C-14), 151.4 (C-2), 153.0 (C-5 or C-8), 154.0 (C-8 or C-5), 183.3 (C-1 or C-4), 184.5 (C-4 or C-1); HRESIMS *m/z *[M+H]^+^= 301.1450 (error 3.373 ppm; calc to C_18_H_21_O_4 _*m/z *301.1439).

### Antibacterial activity 

#### Bacteria

Antibacterial activity was tested against five gram positive bacteria (*Bacillus megaterium*, *Enterococcus faecalis*, *Microbacterium arborescens*, *Micrococcus luteus* and *Staphylococcus epidermidis*) and five gram negative bacteria (*Citrobacter koseri*, *Hafnia alvei*, *Pseudomonas proteolytica*, *Stenotrophomonas maltophilia* and *Yersinia intermedia*). The tested bacteria (as clinical isolates; confirmed via MALDI TOF MS Biotyper, Bruker Daltonics, Billerica, USA) were provided from a collection of Department of Microbiology, Faculty of Biotechnology and Food Sciences, Nitra, Slovakia.

#### Suspension preparation 

All microorganisms were kept on -80 °C. Cultures of bacteria were incubated on Mueller Hinton agar (Merck, Germany) for 24 h at 36 °C. Regulation of initial suspension turbidity was conducted by comparison with 0.5 McFarland's standard (Andrews, 2005[2]). Initial bacterial suspensions contained about 10^8^ CFU/mL. In addition, 1:100 dilutions of initial suspension were made into sterile 0.85 % NaCl. 

#### Micro-dilution antibacterial assay 

Minimal inhibitory concentrations (MICs) were determined by the microbroth dilution method according to the Clinical and Laboratory Standards Institute document (CLSI, 2009[6]) in Mueller Hinton broth (Biolife, Italy). Briefly, the DMSO solutions of isolated naphthoquinones (or standard antibiotics) were prepared as serial two-fold dilutions with the final concentrations ranging between 0.25-512 μg/mL. After that, each well was inoculated with microbial suspension at the final density of 0.5 McFarland. After 24 h of incubation at 37 °C, the inhibition of microbial growth was evaluated by measuring the well absorbance at 450 nm in an absorbance microplate reader Biotek EL808 with shaker (Biotek Instruments, USA). The 96-microtiter plates were read before and after experiment. Measurement error was established for 0.05 values of absorbance. Wells without tested naphthoquinones (or standard antibiotics) were used as negative controls of growth. Pure DMSO was used as a negative control. This experiment was done in eight-replicates for a higher accuracy of the MICs of tested compounds. 

#### Statistical analysis for antibacterial activity

Differences in absorbance between the measurements before and after the analysis were expressed as a set of binary values. These values were assigned to exact concentrations. The following formula was created for this specific experiment: value 1 (inhibitory effect) was assigned to absorbance values lower than 0.05, while value 0 (no effect or stimulant effect) was assigned to absorbance values higher than 0.05. For this statistical evaluation the probit analysis in Statgraphics software was used. 

### Cytotoxicity

#### Drugs and chemicals

Tested isolated naphthoquinones were diluted in DMSO at the concentration of 200 μg/mL, filtered through a 0.22 μm Millipore filter before use, and diluted by a cell culture medium to diverse working concentrations, so that the final concentration of DMSO in cell culture medium never exceeded 0.5 % (v/v).

#### Cell lines

Human breast carcinoma cell line MDA-MB-231 and colon cancer cell line HCT116 were purchased from the American Type Culture Collection (ATCC, USA) and cultured as suggested by the supplier. Both cell lines were sustained in culture medium supplemented with 10 % heat-inactivated FBS. 

#### MTT cell viability assay

MDA-MB-231 and HCT116 cells were plated onto microtiter plates at density of 5 x 10^4^/mL and allowed to adhere overnight. Cells were then treated with tested substances at indicated concentrations, or with media alone (control). The plates were incubated for 24, 48 and 72 h at 37 °C in an atmosphere of 5 % CO_2_ and absolute humidity. The media was removed and 100 μL of MTT (0.5 mg/mL PBS) was added to each well. After 4h incubation at 37 °C, MTT solution was removed and 150 μL of DMSO was added to dissolve the formazan crystals. Absorbance was measured with a multiplate reader (Zenith 3100, Anthos Labtec Instruments GmbH, Austria) at 595 nm (Mosmann, 1983[16]). The IC_50_ values were calculated using Microsoft Office Excel free add-in (ed50v10.xls.) for linear regression downloaded from http://www.sciencegateway.org/protocols/cellbio/drug/data/.

#### Cell cycle analysis

MDA-MB-231 and HCT116 cells were incubated with media alone (control) or with 100 μg/mL of compounds ***1***-***5*** and 500 μg/mL of compounds ***6*** and ***7***. After 48 h at 37 °C in an atmosphere of 5 % CO_2_ and absolute humidity, both attached and detached cells were collected, washed in PBS and fixed with 70 % ethanol at 4 °C. Fixed cells were washed in PBS and finally resuspended in 1mL PBS containing RNAse A (500 μg/mL). After 30 min incubation at 37 °C, cells were stained with 5 μL PI (10 mg/mL PBS), incubated for 15 min in the dark and immediately analyzed by flow cytometer Cytomics FC500 (Beckman Coulter, USA). Distribution of cells with different DNA content was determined with Flowing Software (http://www.flowingsoftware.com/) and presented by histograms. 

#### Determination of apoptosis/necrosis by flow cytometry

Apoptosis was determined using Annexin V-FITC/7-AAD Kit. MDA-MB-231 and HCT116 cells were incubated with media alone (control) or with 100 μg/mL of compounds ***1***-***5*** and 500 μg/mL of compounds ***6*** and ***7***. After 48 h incubation at 37 °C in the atmosphere containing 5 % CO_2_, both attached and detached cells were collected. After washing with PBS, cells were resuspended in 500 μL of ice cold binding buffer. Ten μL of Annexin V-FITC and 20 μL of 7-AAD were added and after 15 min incubation in the dark, 400 μL of binding buffer was added to each tube. Samples were analyzed by flow cytometer Cytomics FC500 (Beckman Coulter, USA). Data were analyzed using Flowing Software and presented by dot plots. 

## Results and Discussion

### Isolation and structural analysis of naphthoquinone derivatives from the roots of O. visianii

**Figure 1:**
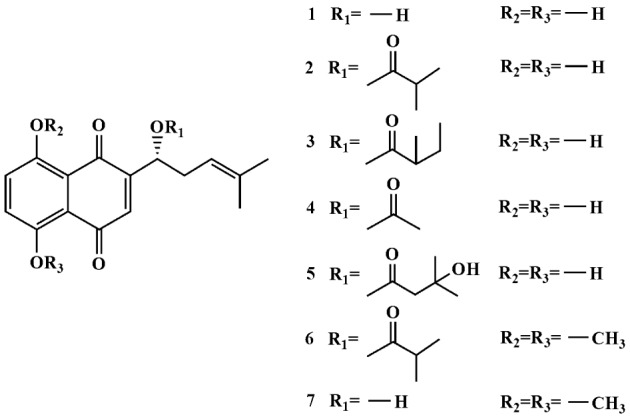
Chemical structures of isolated naphthoquinones *1*-*7*

Separation of petroleum ether-methylene chloride root extract of *O. visianii* using open column chromatography, preparative thin layer chromatography, as well as semipreparative HPLC, resulted in isolation of seven pure naphthoquinone derivatives: deoxyshikonin (***1***) (19 mg), isobutyrylshikonin (***2***) (38 mg), α-methylbutyrylshikonin (***3***) (63 mg), acetylshikonin (***4***) (166 mg), β-hydroxyisovalerylshikonin (***5***) (131 mg), 5,8-*O*-dimethyl isobutyrylshikonin (***6***) (18 mg) and 5,8-*O*-dimethyl deoxyshikonin (***7***) (32 mg) (Figure 1(Fig. 1)). Structural identification of all isolated naphthoquinones was performed by spectroscopic techniques listed in experimental part, as well as by comparison of obtained data with those previously published in the literature (Kretschmer et al., 2012[14]; Ozgen et al., 2004[20]; Albreht et al., 2009[1]; Wang et al., 2015[26]; Zhou et al., 2010[27]). Among isolated compounds, 5,8-*O*-dimethyl isobutyrylshikonin (***6***) was found in nature for the first time.

The compounds ***1***-***5*** showed IR absorptions in the region of 3680-3160 cm^-1^ which indicated presence of OH groups attached to naphthoquinone moiety, as well as tertiary OH group from hydroxyisovaleryl part of compound ***5***. Except in the cases of ***1*** and ***7***, observed stretching vibrations of compounds ***2***-***6*** in the region of 1742-1732 cm^-1^ belong to the aliphatic ester C=O groups. Also, for all isolated naphthoquinones (***1***-***7***), strong bands from C-O groups were noted (at 1278-1018 cm^-1^). The ^1^H NMR spectra of ***1***-***5*** showed two deshielded protons from hydroxyl groups attached to C-5 and C-8 carbons from naphthoquinone part (12.41 ppm-12.64 ppm). Also, compound ***5*** contains one additional broadened singlet (at 3.26 ppm) from hydroxyl group linked to C-3'. Instead of singlets of OH protons, resonances from C-5-OCH_3_ and C-8-OCH_3 _groups (singlets at 3.95 ppm and 3.96 ppm, respectively) were observed in the ^1^H NMR spectra of ***6*** and ***7*** (as dimethoxy derivatives). In contrast to other isolated compounds (for ***2***-***6***, doublet of doublets in the region of 6.02 ppm-6.10 ppm), deoxyshikonin ***1*** and it 5,8-dimethoxy derivative ***7*** showed signals of C-11-H protons at lower fields (multiplets at 2.28 ppm and 2.52 ppm, respectively). All derivatives contain characteristic triplets from vinyl C-13-H protons (at 5.12 ppm-5.13 ppm), singlets from C-3-H protons (naphthoquinone moiety; at 6.60 ppm-7.03 ppm), as well as singlets from C-H-6 and C-H-7 protons (naphthoquinone moiety; at 7.18 ppm-7.31 ppm). In the ^13^C NMR spectra of ***2***-***6***, signals from C-1' carbons (at 169.5 ppm-175.7 ppm) were recorded, indicating the presence of aliphatic ester C=O groups. These signals were not observed in cases of compounds ***1*** and ***7***, as deoxyshikonin and its dimethoxy derivative. Also, in contrast to isolated compounds ***2*** and ***1***, 5,8-dimethoxy derivatives ***6*** and ***7*** showed two additional signals from C-5-OCH_3_ and C-8-OCH_3_ carbons (56.8 ppm and 56.9 ppm, respectively). Finally, the positive values of measured optical rotations ([α]_D_^25^) confirmed that isolated naphthoquinones ***2***-*6* from the roots of *O*. *visianii* are shikonin derivatives.

### In vitro activity of the isolated naphthoquinones against bacteria 

The seven isolated naphthoquinones were evaluated for their antibacterial activity against the five gram positive and five gram negative bacteria (Table 1(Tab. 1)). Resistance of used bacteria was presented in Table 1(Tab. 1), while the activities of isolated compounds (as MIC_50_ and MIC_90_ in μg/mL, respectively) were showed in Table 2(Tab. 2). Observed MIC_50_ and MIC_90_ values of isolated naphthoquinones are in the range of 4.27 μg/mL-68.27 μg/mL and 4.77 μg/mL-76.20 μg/mL, respectively. In accordance with literature (Noundou et al., 2016[19]), compounds which exhibited MIC values equal or less than 16 μg/mL were considered to have possible clinical relevance. Following these facts, erythromycin, ampicillin and vancomycin resistant *B. megaterium* is the most sensitive bacteria against tested compounds ***1***, ***3*** and ***4***, ampicillin and vancomycin resistant *E. faecalis* against compounds ***3***, ***4*** and ***5***, while other three gram positive bacteria are sensitive against ***3*** and ***4***. Among gram negative bacteria, meropenem resistant* S. maltophilia* is susceptible to ***2***, ***3***, and ***4***, while other four bacteria showed high sensitivity against compounds ***3*** and ***4***. Previously published data indicated notable activity of shikonin derivatives against gram positive bacteria (and generally low activity against gram negative bacteria), with the fact of remarkable differences of antibacterial potency due to different approaches of measuring MIC values (Papageorgiou et al., 1999[23]; Andujar et al., 2013[3]). On the other hand, some results showed good antibacterial activity against some gram negative species (Brigham et al., 1999[4]; Ding et al., 2011[9]). Our results indicated that isolated naphthoquinone derivatives at the same time showed a good activity towards both gram positive and gram negative bacteria. 

Among the isolated compounds, α-methylbutyrylshikonin 3 and acetylshikonin 4 exhibited the highest antibacterial activities toward all tested bacterial species. Observed MIC50 and MIC90 values for gram positive bacteria are in the range of 6.40 µg/mL-12.79 μg/mL and 6.82 μ g/mL-13.60 μg/mL, respectively, while for gram negative bacteria are in the range of 4.27 μg/mL-8.53 μg/mL and 4.77 μg/mL-9.54 μg/mL, respectively. The shikonin derivative 3 had strong potency against *E. faecalis* (MIC50 6.40 μg/mL and MIC90 6.82 μg/mL), as well as against *S. maltophilia* (MIC50 4.27 μg/mL and MIC90 4.77 µg/mL), while compound 4 was very active against *M. arborescens* (MIC50 6.40 μg/mL and MIC90 6.82 μg/mL) and *P. proteolytica* (MIC50 4.27 μg/mL and MIC90 4.7 μg/mL). In contrast to previous report that alkylation of the phenolic groups leads to lower activity (Papageorgiou et al., 1999[23]), we herein reported that gram negative ampicillin resistant bacteria *Y. intermedia* was very susceptible against compound 7 as dimethoxy derivative of deoxyshikonin (MIC50 12.79 μg/mL and MIC90 13.60 μg/mL).

From the above mentioned results, in cases of developed resistance (or in cases of preventing resistance) examined naphthoquinones may substitute clinical antibiotics.

### Cytotoxic activity of isolated naphthoquinones

**Figure 2:**
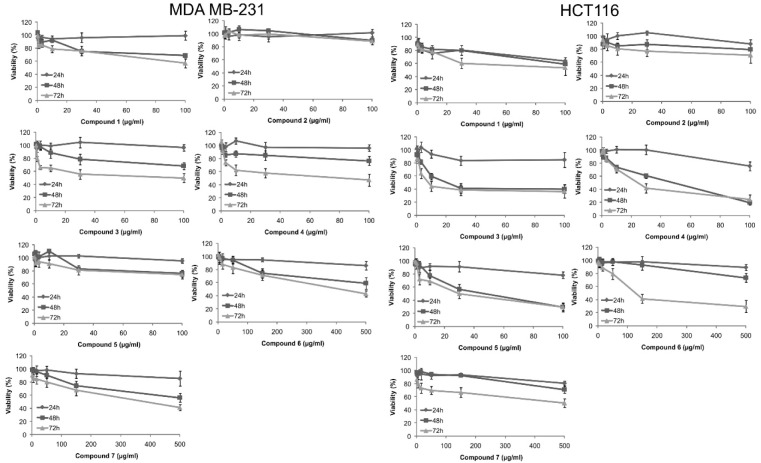
Cell viability of MDA-MB-231 and HCT116 cell lines after 24 h, 48 h and 72 h treatment with diverse concentrations of tested naphthoquinones, estimated by MTT assay (results from three separate experiments; mean value ± SD)

Cytotoxic activity of compounds ***1-7*** against two cancer cell lines was performed by conducting MTT cell viability assays. Generally, tested compounds decreased viability of both tumor cell lines in a dose and time dependent manner. Yet, the extent of cytotoxicity on the designated cell lines was diverse, being more potent on HCT116 cell line (Figure 2(Fig. 2), Table 3(Tab. 3)).

As observed in Table 3(Tab. 3), α-methylbutyrylshikonin ***3*** and acetylshikonin ***4*** exhibited the highest cytotoxic activity against MDA-MB-231 cells compared to all other tested substances with (IC_50_ values 86.0 μg/mL and 80.2 μg/mL, respectively). Also, all examined compounds, except compounds ***6*** and ***7***, decreased viability of MDA-MB-231 cells after 48 hours of incubation. However, HCT116 cells were generally more sensitive to the effects of isolated naphthoquinones. Compound ***3*** showed the strongest cytotoxic effect against HCT116 cells with IC_50_ value of 15.2 μg/mL. Also, compounds ***1***, ***4*** and ***5*** significantly decreased viability of HCT116 cells and these compounds exhibited IC_50_ values of 97.8 μg/mL, 24.6 μg/mL and 30.9 μg/mL, respectively. The results presented in Table 3(Tab. 3) are in accordance with the previously published data of cytotoxicity of shikonin derivatives against many cancer cell lines. Among 1500 tested quinones, about 150 compounds showed strong *in vivo* activity against W256 in rats and P388 lymphoid leukemia in mice, as well as significant *in vitro* potency toward KB cells and CCRF-CEM leukemia cells (Papageorgiou et al., 1999[23]). On the other hand, isolated quinones demonstrated significant *in vitro* potency toward KB cells, CCRF-CEM leukemia cells, as well as some melanoma cell lines (Papageorgiou et al., 1999[23]; Kretschmer et al., 2012[14]). To determine whether cytotoxicity induced by naphthoquinones was due to apoptosis or cell cycle arrest, the cells were stained with fluorescent dyes and analyzed on flow cytometer.

### Naphthoquinones induce apoptosis in tumor cell lines

**Figure 3:**
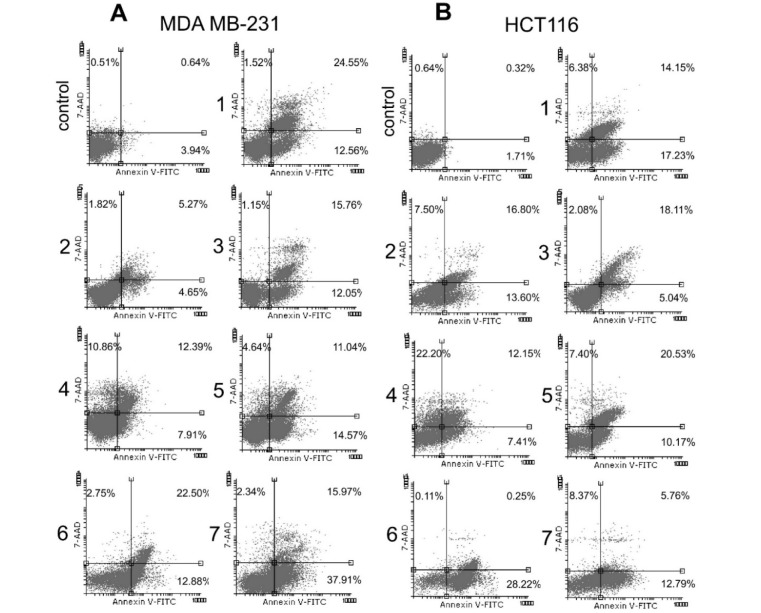
Flow cytometric analysis of Annexin V-FITC/7-AAD stained MDA-MB-231 (A) and HCT116 (B) cells (The percentages of early apoptotic (Annexin V+7-AAD-, lower right quadrant), late apoptotic (Annexin V+7-AAD+, upper right quadrant) and necrotic cells (Annexin V-7-AAD+, upper left quadrant) in untreated and treated cells are indicated on dot plots)

Flow cytometric analysis showed that primary type of cell death in MDA-MB231 cells, induced by isolated naphthoquinones, was apoptosis, with small proportion of necrotic cells, whereas percent of necrotic cells was considerable higher only in cells treated with compound ***4*** (Figure 3(Fig. 3)). 

Similarly, in HCT116 cells, the supreme type of cell death induced by naphthoquinones was apoptosis and again, only compound ***4*** induced similar percent of both apoptosis and necrosis (Figure 3(Fig. 3)). The scope of cancer therapy is to promote apoptosis, a type of cell death that is, unlike necrosis, limited to cells committed to die and does not affect surrounding tissue. Therefore, agents that induce apoptosis in cancer cells can be useful in cancer treatment. 

### Naphthoquinones induce cell cycle arrest in tumor cell lines

**Figure 4:**
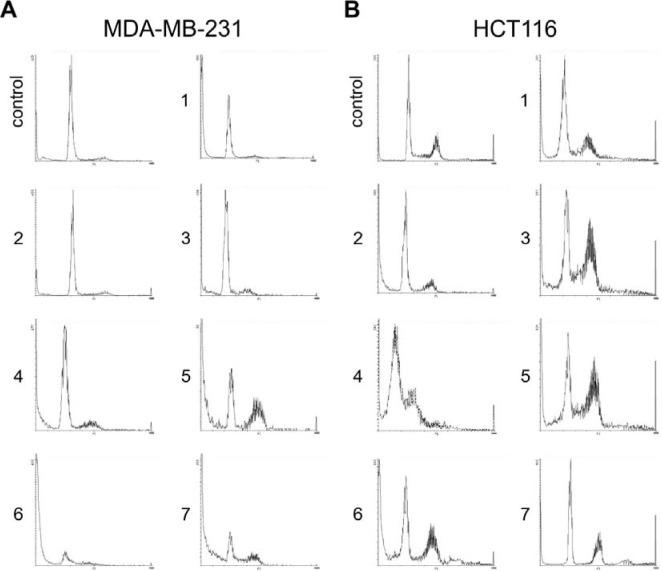
Flow cytometric analysis of cell cycle (histograms present cell cycle distribution in untreated and treated MDA-MB-231 (A) and HCT116 (B) cells)

A 48 hour treatment of MDA-MB-231 cells with compounds ***3***-***7*** resulted in significant increase in percentage of the cells assembled in G2/M phase, with concomitant decrease in percentage of cells in G0/G1 phase (p < 0.05) (Figure 4(Fig. 4)). 

**Figure 5:**
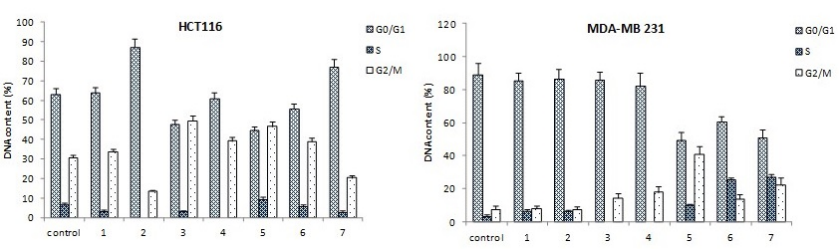
Effect of naphthoquinones on cell cycle (bars present cell cycle distribution in untreated and treated MDA-MB-231 (A) and HCT116 (B) cells)

The percentage of MDA-MB-231 cells batched in G2/M phase increased from 7.6 % in control to 14.0 % - 40.8 % in treated cells. Additionally, compared to control, compounds ***5***, ***6*** and ***7*** showed a significant rise in the number of the cells arrested at S phase (3.2 %, 10.1 %, 25.4 % and 27.1 % respectively). Compounds ***1*** and ***2*** showed no changes in cell cycle profile of MDA-MB-231 cells (Figure 4(Fig. 4)). Similarly, treatment of HCT116 cells with compounds ***3***, ***4***, ***5*** and ***6*** resulted in increase of G2/M phase cells from 30.7 % (control) to 38.9 %-49.6 %, with most pronounced arrest when treated with compound ***3*** (p < 0.05). Contrary, treatment with compounds ***2*** and ***7*** showed an increasing trend in the number of the cells arrested at G0/G1 phase: quantitatively it was 24.3 % and 14.7 % respectively over the control values. Compound ***1*** showed no changes in cell cycle profile of HCT116 cells (Figure 4(Fig. 4) and Figure 5(Fig. 5)).

Physiologically, cell cycle and apoptosis are directly connected. In damaged cells, stopping the cell cycle, gives cells a time to activate repair mechanisms and fix the damage. If the damage can't be fixed, apoptosis program is activated. Contrary, uncontrolled progression of the cell cycle and avoidance of apoptosis are the main characteristics of malignant cells (Hanahan and Weinberg, 2000[12]). Our results demonstrated that naphthoquinones arrest mitosis and make the cell cycle stopped in G0/G1, S or G2/M phases resulting in cellular apoptosis of target cells. The difference between cytotoxic effects of isolated naphthoquinones on selected tumor cell lines, was probably caused by different molecular targets of these compounds (Narang and Desai, 2009[17]). Literature reported that activity of naphthoquinones was closely related to do presence of quinone part, with the facts that mode of cytotoxic action includes oxidative stress, as well as inhibition of topoisomerase I and topoisomerase II (Papageorgiou et al, 1999[23]). Thus, G0/G1 arrest in HCT116 (compounds ***2*** and ***7***) or S phase arrest in MDA-MB-231 (compounds ***6*** and ***7***) point to blockade of DNA replication and inhibition of topoisomerase II and I as a possible mechanism that triggers apoptosis (Huang et al., 2003[13]), whereas G2/M arrest coupled with decreased expression of cyclin-dependent kinases 1 and 2 with contribution from ROS-mediated pathways (Pan et al., 2015[21]) may be responsible for apoptosis of MDA-MB-231 cells induced by compounds*** 3***-***7***.

## Conclusions

In this study, for the first time we presents that roots of *O. visianii* are a rich source of bioactive naphthoquinone derivatives which possess strong activity toward selected resistant bacterial strains, as well as high cytotoxic activity against MDA-MB-231 and HCT116 tumor cell lines. According to our results, α-methylbutyrylshikonin ***3*** and acetylshikonin ***4*** showed notable activities toward all tested gram positive and gram negative bacterial species. Also, compounds ***3*** and ***4*** exhibited strong cytotoxic potency against MDA-MB-231 cells, while compounds ***3, 4 ****and**** 5*** showed the strongest cytotoxic effect toward HCT116 cells. Overall, this research provided a strong indication of the potential use of *O. visianii* naphthoquinone pigments as antibacterial and cytotoxic agents. 

## Acknowledgement

This work was financially supported by the Ministry of Education, Science and Technological Development of the Republic of Serbia under Grants III41010, OI 172053, and co-funded by VEGA project No. 1/0611/14.

## Figures and Tables

**Table 1:**
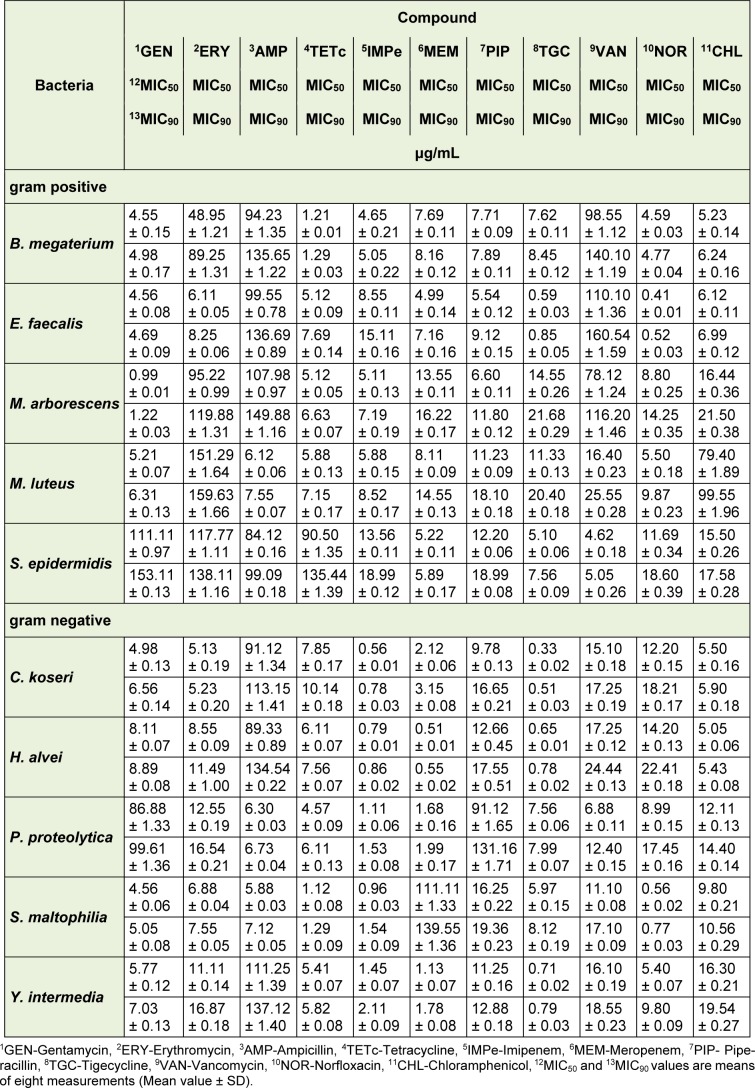
Antibacterial activity of tested antibiotics

**Table 2:**
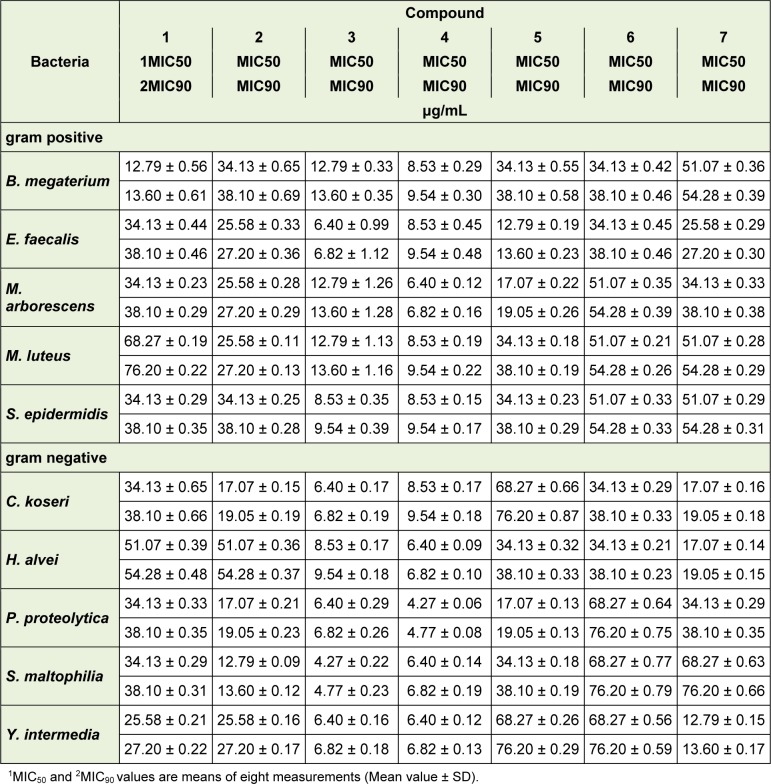
Antibacterial activity of tested naphthoquinones

**Table 3:**
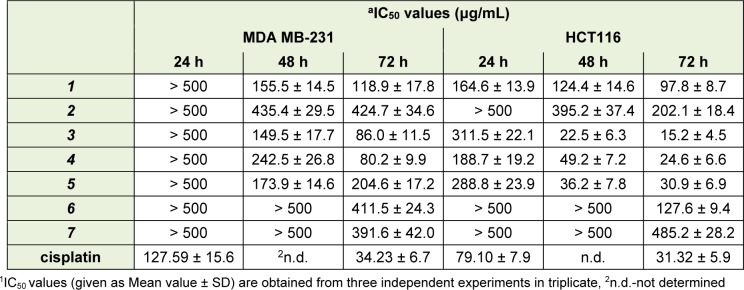
IC_50_ (μg/mL) values determined by MTT assay for investigated compounds, after 24 h, 48 h and 72 h treatment of MDA-MB-231 and HCT116 cells

## References

[1] Albreht A, Vovk I, Simonovska B, Srbinoska M (2009). Identification of shikonin and its ester derivatives from the roots of Echium italicum L. J Chromatogr A.

[2] Andrews JM (2005). BSAC standardized disc susceptibility testing method (version 4). J Antimicrob Chemother.

[3] Andujar I, Rios JL, Giner RM, Recio MC (2013). Pharmacological properties of shikonin - A review of literature since 2002. Planta Med.

[4] Brigham LA, Michaels PJ, Flores HE (1999). Cell-specific production and antimicrobial activity of naphthoquinones in roots of Lithospermum erythrorhizon. Plant Physiol.

[5] Cadirci E, Suleyman H, Aksoy H, Halici Z, Ozgen U, Koc A (2007). Effects of Onosma armeniacum root extract on ethanol-induced oxidative stress in stomach tissue of rats. Chem Biol Interact.

[6] CLSI, Clinical and Laboratory Standard Institute (2009). Clinical and Laboratory Standards Institute CLSI Document: Performance standard for antimicrobial susceptibility testing;informational supplement M100-S21.

[7] Damianakos H, Sotiroudis G, Chinou I (2013). Pyrrolizidine alkaloids from Onosma erecta. J Nat Prod.

[8] Davis PH (1988). Flora of Turkey and the East Aegean Islands. Vol 6.

[9] Ding X, Yin B, Qian L, Zeng Z, Yang Z, Li H (2011). Screening for novel quorum-sensing inhibitors to interfere with the formation of Pseudomonas aeruginosa biofilm. J Med Microbiol.

[10] El-Shazly A, Abdel-Ghani A, Wink M (2003). Pyrrolizidine alkaloids from Onosma arenaria (Boraginaceae). Biochem Sys Ecol.

[11] Gyles C (2011). The growing problem of antimicrobial resistance. Can Vet J.

[12] Hanahan D, Weinberg RA (2000). The hallmarks of cancer. Cell.

[13] Huang X, Traganos F, Darzynkiewicz Z (2003). DNA damage induced by DNA topoisomerase I- and topoisomerase II-inhibitors detected by histone H2AX phosphorylation in relation to the cell cycle phase and apoptosis. Cell Cycle.

[14] Kretschmer N, Rinner B, Deutsch AJ, Lohberger B, Knausz H, Kunert O (2012). Naphthoquinones from Onosma paniculata induce cell-cycle arrest and apoptosis in melanoma Cells. J Nat Prod.

[15] Mellidis AS, Papageorgiou VP, Kokkalou E (1993). Phenolic constituents from Onosma heterophylla. J Nat Prod.

[16] Mosmann T (1983). Rapid colorimetric assay for cellular growth and survival: application to proliferation and cytotoxicity assays. J Immunol Methods.

[17] Narang AS, Desai DS, Lu Y, Mahato RI (2009). Anticancer drug development. Pharmaceutical perspectives of cancer therapeutics.

[18] Naz S, Ahmad S, Ajaz Rasool S, Asad Sayeed S, Siddiqi R (2006). Antibacterial activity directed isolation of compounds from Onosma hispidum. Microbiol Res.

[19] Noundou XS, Krause RW, van Vuuren SF, Ndinteh DT, Olivier DK (2016). Antibacterial effects of Alchornea cordifolia (Schumach. and Thonn.) Müll. Arg extracts and compounds on gastrointestinal, skin, respiratory and urinary tract pathogens. J Ethnopharmacol.

[20] Ozgen U, Coskun M, Kazaz C, Secen H (2004). Naphthoquinones from the Roots of Onosma argentatum Hub.-Mor. (Boraginaceae). Turk J Chem.

[21] Pan ST, Qin Y, Zhou ZW, He ZX, Zhang X, Yang T (2015). Plumbagin induces G2/M arrest, apoptosis, and autophagy via p38 MAPK- and PI3K/Akt/mTOR-mediated pathways in human tongue squamous cell carcinoma cells. Drug Des Develop Ther.

[22] Papageorgiou VP, Assimopoulou AN, Ballis AC (2008). Alkannins and shikonins: a new class of wound healing agents. Curr Med Chem.

[23] Papageorgiou VP, Assimopoulou AN, Couladouros EA, Hepworth D, Nicolaou KC (1999). The chemistry and biology of alkannin, shikonin, and related naphthazarin natural products. Angew Chem Int Edit.

[24] Sezik E, Yesilada E, Tabata M, Honda G, Takaishi Y, Fujita T (1997). Traditional medicine in Turkey VIII. Folk medicine in east anatolia;Erzurum, Erzincan, Agri, Kars, Igdir provinces. Econ Bot.

[25] Shcherbanovskii LR (1971). Onosma visianii-A new source of shikonin. Chem Nat Compd.

[26] Wang L, Li F, Liu X, Chen B, Yu K, Wang MK (2015). Meroterpenoids and a naphthoquinone from Arnebia euchroma and their cytotoxic activity. Planta Med.

[27] Zhou W, Peng Y, Li SS (2010). Semi-synthesis and anti-tumor activity of 5,8-O-dimethyl acylshikonin derivatives. Eur J Med Chem.

